# Too many Japanese university students are still smoking tobacco

**DOI:** 10.1186/1617-9625-4-10

**Published:** 2008-11-18

**Authors:** Derek R Smith, Ken Takahashi

**Affiliations:** 1 WorkCover New South Wales Research Centre of Excellence, Faculty of Health, University of Newcastle, Ourimbah, Australia; 2 Department of Environmental Epidemiology, University of Occupational and Environmental Health, Kitakyushu, Japan

## Abstract

Although campus-wide smoking bans are slowly spreading throughout Japan, the uptake of these measures has been suboptimal and many Japanese university students continue to smoke. Educational facilities are in an ideal position to set positive examples for tobacco control, and the time is now right for more Japanese universities to actively encourage their students as health promotion advocates and role models for healthy behavior.

## Main text

Japanese society represents an ongoing challenge for antismoking movements. The domestic market is dominated by Japan Tobacco, of which the government owns a share [1] and is legally obliged to promote cigarette sales [2]. Large multinational corporations are also present, with innovative and culturally-specific techniques such as the 'clean cigarette' for a clean nation [3], among others. Tobacco taxes are relatively low by international standards, allowing Japan to provide some of the most affordable cigarettes in the world [4]. Smoking rates among Japanese men have remained high, while the prevalence among young women has also been increasing in recent years. By 2003 however, lung cancer had begun exceeding stomach cancer as the leading cause of mortality [2]. Some national cohort studies have also shown that the life expectancy of Japanese decreases as their level of smoking increases [5]. Despite these facts, many Japanese health care workers and their student counterparts continue to smoke tobacco at relatively high rates when compared internationally.

Tobacco use among health care students has been recently reviewed from a global perspective, with high smoking rates being documented in Japanese medical, dental and nursing faculties [6-8]. A survey of Japanese university students was also conducted by the *Ministry of Health, Labor and Welfare *(MHLW) in 2007 [9], which showed that dental students had the highest smoking rates across health science faculties, with 62% of male and 35% of females being current smokers. Their tobacco usage exceeded national averages for the same age group, where around 49% of men and 19% of women in their 20s, smoked [9]. Interestingly, it was also higher (among males) than an antecedent study of dental students in the United Kingdom, during which 54% of male and 40% of female students used tobacco products [10], even though the latter research had been conducted in the early 1960s. Cultural differences are important to consider however, as research in the early 1990s suggested, for example, that more Irish dental students might smoke when compared to their Canadian counterparts [11]. Contemporary studies also suggest that differences in dental students' smoking prevalence rates by country, still persist [12-14].

Although students should clearly be aware of their health promotion role, the aforementioned Japanese MHLW study revealed that when it came to their patient's smoking habits, most students were comparatively forgiving [9]. This attitude may reflect the intrinsic cultural issues of social harmony and cohesion which prevail in Japan, with passive smoking for example, having long been regarded as a problem of manners, rather than health [3]. Japan ratified the *Framework Convention on Tobacco Control *(FCTC) in 2004 and enacted it during 2005, although this appears to have had only a limited impact on smoking rates [15]. While campus-wide smoking bans are slowly spreading throughout the country, by 2007 such measures had only been adopted or planned by less than half of all medical and dental schools [16].

Given that they are responsible for the next generation of health care workers, educational facilities are in an ideal position to set positive examples in tobacco control. Much can be learned from their international counterparts, and the time is now right for more Japanese universities to actively encourage their students as health promotion advocates. A greater focus on the Ottawa Charter and more aggressive teaching on the adverse health effects of tobacco use would clearly be a positive step towards optimal health for all tertiary students, regardless of nationality.

## Competing interests

The authors declare that they have no competing interests.

## Authors' contributions

DRS conceived the idea for the study. DRS and KT wrote the manuscript. Both authors read and approved the final version of the manuscript.
